# GABA and Glutamate Pathways Are Spatially and Developmentally Affected in the Brain of *Mecp2*-Deficient Mice

**DOI:** 10.1371/journal.pone.0092169

**Published:** 2014-03-25

**Authors:** Rita El-Khoury, Nicolas Panayotis, Valérie Matagne, Adeline Ghata, Laurent Villard, Jean-Christophe Roux

**Affiliations:** 1 Aix Marseille Université, GMGF, Marseille, France; 2 Inserm, UMR_S 910, Marseille, France; 3 Department of Biological Chemistry, Weizmann Institute of Science, Rehovot, Israel; University of Minnesota, United States of America

## Abstract

Proper brain functioning requires a fine-tuning between excitatory and inhibitory neurotransmission, a balance maintained through the regulation and release of glutamate and GABA. Rett syndrome (RTT) is a rare genetic disorder caused by mutations in the methyl-CpG binding protein 2 (*MECP2*) gene affecting the postnatal brain development. Dysfunctions in the GABAergic and glutamatergic systems have been implicated in the neuropathology of RTT and a disruption of the balance between excitation and inhibition, together with a perturbation of the electrophysiological properties of GABA and glutamate neurons, were reported in the brain of the *Mecp2*-deficient mouse. However, to date, the extent and the nature of the GABA/glutamate deficit affecting the *Mecp2*-deficient mouse brain are unclear. In order to better characterize these deficits, we simultaneously analyzed the GABA and glutamate levels in *Mecp2*-deficient mice at 2 different ages (P35 and P55) and in several brain areas. We used a multilevel approach including the quantification of GABA and glutamate levels, as well as the quantification of the mRNA and protein expression levels of key genes involved in the GABAergic and glutamatergic pathways. Our results show that *Mecp2*-deficient mice displayed regional- and age-dependent variations in the GABA pathway and, to a lesser extent, in the glutamate pathway. The implication of the GABA pathway in the RTT neuropathology was further confirmed using an *in vivo* treatment with a GABA reuptake inhibitor that significantly improved the lifespan of *Mecp2*-deficient mice. Our results confirm that RTT mouse present a deficit in the GABAergic pathway and suggest that GABAergic modulators could be interesting therapeutic agents for this severe neurological disorder.

## Introduction

The mammalian brain requires a balance between excitatory and inhibitory neurotransmission to sustain proper neuronal function [1]. Among the many CNS neurotransmitters, biogenic amines and amino-acids are particularly important. Two major classes of neurons maintain this intricate dialogue: the excitatory projecting neurons and their local inhibitory neighbors. In adulthood, excitation is prominently mediated by the neurotransmitter glutamate while γ-aminobutyric acid (GABA) has an inhibitory effect. Given the regulatory roles of GABA and the importance of a balanced, fine-tuned signaling between excitatory and inhibitory cells, it is not unexpected that dysfunctions in GABA neurons and GABA-regulated circuits have been implicated in several psychiatric and neurological diseases [2]–[4], including Rett syndrome (RTT). RTT is caused by mutation in the methyl-CpG binding protein 2 gene (*MECP2*) [5], [6]. Previous studies in RTT have shown impairment in the integration of neurochemical inputs, fundamental for information processing. In particular, biogenic amines deficits have been reported and extensively studied both in patients and *Mecp2*-deficient (*Mecp2* ^-/y^) mice [7]–[15]. Dysfunctions in the GABAergic and glutamatergic systems have also been pointed out since the balance between brain excitation and inhibition is impaired in the brain of the *Mecp2*-deficient mouse [16]–[19]. Moreover, Mecp2 differentially regulates the maturation of GABAergic synapses in excitatory and inhibitory neurons in the thalamus [20]. However, contrary to biogenic amines, there does not seem to be a clear-cut pattern in the way the GABA-Glutamate network is affected and some authors have reported over-excitation or -inhibition for the same brain area. Nevertheless, in adult mice, excitatory synaptic transmission seems to be increased in the principal brainstem nuclei [21]–[24], as well as in the hippocampus [16], [25]–[29], whereas a decrease is reported in sensory and motor-frontal cortices [18], [19], [30], [31]. More recently, Kron *et al* [32] used the immediate-early gene c-Fos to map the neural activity in several brain regions of the *Mecp2*-deficient mice. Their results revealed a striking reduction in c-Fos expression in the midbrain of *Mecp2*-deficient mice while and an increase in activity was observed in the brainstem of the same mice. These changes could be due, in part, to a regional variation in the GABA/glutamate ratio since both neurotransmitters are the main inhibitory and excitatory brain mediators. Finally, the combination of a GABA reuptake blocker with a serotonin-1a agonist was proven to be beneficial as it offset breathing defects and lengthened the lifespan of *Mecp2* heterozygous mice [25]. Altogether these results indicate that GABA and/or glutamate dysfunction could play a key role in the appearance of the RTT phenotype. However, no data has yet shown whether this disorder results from a global or a specific neuronal subpopulation dysfunction. Given the importance of the GABA and glutamate for the activity of neuronal networks, it is surprising that only a few studies assayed these amino-acids in the brain of *Mecp2*-deficient mice. Those studies analyzed GABA and glutamate metabolism in whole brains or in a limited number of brain areas in *Mecp2*-deficient animals. In the present study, we report the simultaneous GABA/glutamate variations in *Mecp2*-deficient mice at two critical ages, P35 (pathology onset) and P55 (late disease stage) in several brain areas. We first quantified the GABA and glutamate levels using automatic HPLC coupled to a fluorescence detector. Next, we analyzed the changes in mRNA and protein levels of key genes involved in the GABAergic and glutamatergic pathways. The results show regional- and age–dependent variations in the GABA and glutamate pathways in *Mecp2*-deficient mice compared to their wild type littermates. Finally, an *in vivo* treatment with a GABA reuptake inhibitor significantly improved the lifespan of *Mecp2*-deficient mice suggesting that the modulation of the GABAergic pathway may be a new therapeutic target for the treatment of RTT-associated clinical signs.

## Materials and Methods

### Ethics Statement

The experimental procedures were carried out in compliance with the European guidelines for the care and use of laboratory animals (EU directive 2010/63/EU) and in accordance with the recommendations provided by the guide for the care and use of the laboratory animals of the French national institute for science and health (INSERM). All research involving animals has been specifically approved by the ethical committee of the Aix-Marseille University and the regional committee of the Region Provence-Alpes-Côte d'Azur (Permit Number: 13-405). All experiments were made to minimize animal suffering.

### Animals

Experiments were performed on the B6.129P2(c)- *Mecp2^tm1-1Bird^* RTT mouse model [33]. The mice were obtained from the Jackson Laboratories and maintained on a C57Bl/6 background in the animal facility of the Faculté de Médecine de La Timone, Aix Marseille Université (Marseille, France). A total number of 31 *Mecp2* ^-/y^ and 33 WT male mice were used in this study except for the tiagabine treatment. *Mecp2* ^-/y^ mice were compared to their respective WT littermates. Breeding and genotyping were performed as previously described [33], [34]. Both pre-symptomatic and symptomatic mice were analyzed at 35 and 55 days of age (P35, P55), according to the gradual manifestation of the *Mecp2* ^-/y^ phenotype [35], [36].

### Chemicals

Naphthalene-2,3-dicarboxaldehyde (NDA) and sodium cyanide (NaCN) were purchased from Fluka (Buchs, Switzerland) and acetonitrile from Merck (Darmstadt, Germany). GABA, DL-glutamate (Glu), cysteic acid, boric acid, sodium dodecyl sulfate (SDS), and sodium tetraborate were purchased from Sigma (St. Quentin Fallavier, France), and hydroxypropyl-β-cyclodextrin (HP-β-CD) was obtained from Aldrich (Steinheim, Germany). Standard solutions of 1 mM GABA, Glu, and cysteic acid were stored at 4°C as aliquots in 0.1 M hydrochloric acid (from a 37% stock solution, Merck).

### Tissue sampling

**Figure 1:**
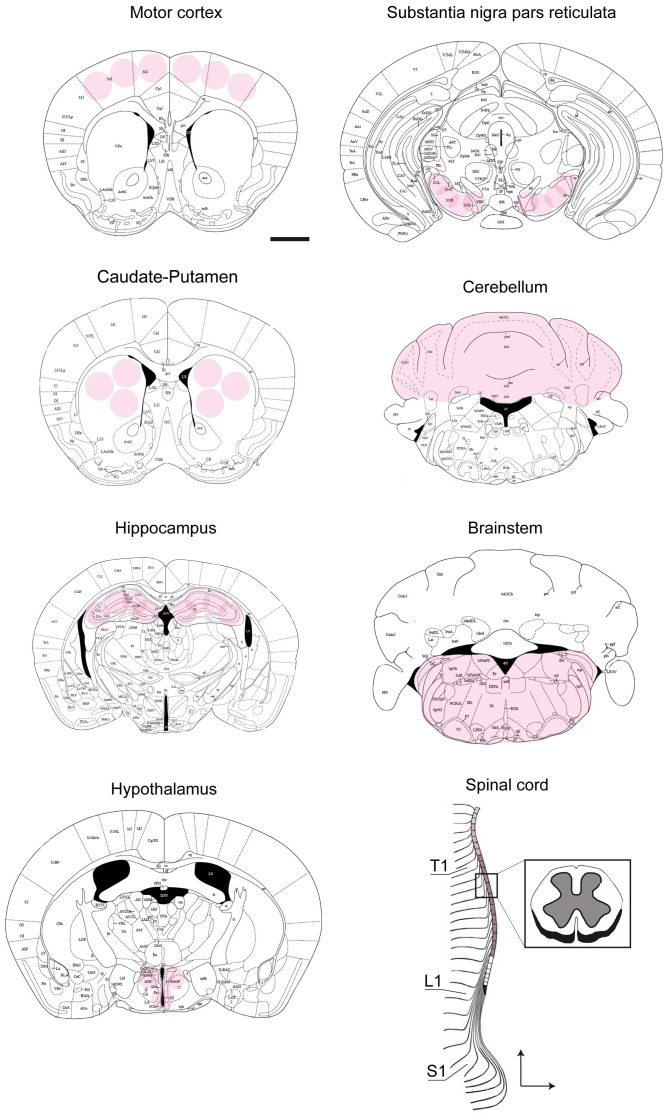
Schematic representation of studied regions. The drawings are adapted from The Mouse Brain in Stereotaxic Coordinates atlas [38]. The shadowed circles/area mark sites where the brain tissue was dissected out. The motor cortex (Distance from the Bregma, +1.18), caudate-putamen (+0.98), hippocampus (−1.94), hypothalamus (−0.82), substantia nigra pars reticulata (−3.28), were micropunched. The brainstem, the cerebellum and the spinal cord were dissected under a binocular microscope. Scale bar, 1 mm.

P35 (n = 9 WT; n = 6 *Mecp2* ^-/y^) and P55 (n = 8 WT; n = 9 *Mecp2* ^-/y^) mice were killed by cervical dislocation and their brains dissected out within the first 2 min post-mortem. The tissue sampling is adapted from the micropunch method described by Palkovits and Brownstein [37]. Brain areas dissection was performed on a cryostat (−20°C) with the help of a 5× magnifying lens, following their stereotaxic coordinates [38] as previously described [9], [10], [39]. The motor cortex (Distance from the Bregma, +1.18), caudate-putamen (+0.98), hypothalamus (−0.82), hippocampus (−1.94) and substantia nigra pars reticulata (SNpr, −3.28) were microdissected using a punching needle (0.5 mm diameter) and kept at −80°C until biochemical analysis. The brainstem and the cerebellum were dissected under a binocular microscope and the whole spinal cord was flushed out of the vertebral canal with ice-cold PBS (Figure 1).

### Tissue extraction for neurochemical dosage

Extraction of microdissected samples was made at 0°C with perchloric acid containing 1.34 mM EDTA and 0.05% w/v sodium bisulfite. Sonication was applied by 20-s periods until homogenization. Extracts were then centrifuged at 14000 rpm at +4°C for 20 min and supernatants were kept frozen until analysis. Extraction volume: 60 µL (motor cortex, caudate-putamen, hippocampus, hypothalamus and SNpr) and 1 ml (cerebellum, brainstem, spinal cord).

### Glutamate and GABA quantification

Glutamate and GABA tissue levels were quantified using an automatic capillary zone electrophoresis P/ACE™ MDQ system (Beckman, USA) equipped with a ZETALIF laser induced fluorescence detector (Picometrics, France) by the core facility Ani-RA Neurochem (Centre de Recherche en Neurosciences de Lyon, France). Excitation was performed using a He–Cd laser (Liconix, USA) at a wavelength of 442 nm, the emission wavelength being 490 nm. The derivatization reagents were added to 10 µL of the diluted extracts (1∶500 in Ringer's solution): borate/NaCN solution (mixing solution [100∶20, v/v] of 500 mM borate buffer pH 8.7, and 87 mM NaCN in water) was delivered at 2 µL each; internal standard (IS) solution (0.1 mM cysteic acid in 0.117 M perchloric acid) and NDA solution (2.925 mM in acetonitrile/water, 50∶50, v/v) were delivered at 1 µL each. Separation was carried out on a 63 cm×50 mm fused-silica capillary (Composite Metal Services, Worcester England) with an effective length of 52 cm. The separation conditions were: an applied voltage of 25 kV, hydrodynamic sample injection (10 s at 0.6 psi) and a temperature between 38 and 41°C. The capillary was sequentially flushed for 30 s with 0.25 M NaOH, ultrapure MilliQ water and running buffer (75 mMol/L sodium borate, pH 9.20±0.02, containing 10 mMol/L HP-β-CD and 70 mM SDS) between analyses. Electropherograms were acquired at 15 Hz using P/ACE™ MDQ software [40].

### RNA extraction and mRNA QPCR

P35 (n = 4 WT; n = 4 *Mecp2*-deficient) and P55 (n = 4 WT; n = 4 *Mecp2*-deficient) mice were killed by cervical dislocation, and their brain areas were dissected out. The caudate-putamen and hippocampal tissues were dissected as previously described for biochemical analysis. The ventral midbrain area (SNpr+substantia nigra pars compacta) was dissected instead of the SNpr alone in order to increase total RNA yield. All samples were kept at −80°C until analysis. Total RNA was extracted using TRIzol reagent (Invitrogen) according to the manufacturer's instructions. RNA samples were treated with 3 U/µL of DNase I, RNase-free (Qiagen) at 37°C during 30 min followed by enzyme inactivation at 65°C during 5 min. The purity of the RNA samples was analysed using the ND-1000 spectrophotometer (NanoDrop, Thermo Scientific) and quality was assessed by electrophoresis on a denaturing agarose gel. Reverse transcription of 2 µg of total RNA was performed in a 20 µl reaction containing 4 µl of FS super script 5× (Invitrogen), 12,5 nM of Random primers, 10 mM dNTP, 0,1 M DTT (Invitrogen), 40 U Rnase out (invitrogen), and 200 U of Superscript II reverse transcriptase (Invitrogen). Quantitative PCR reaction was performed using a LightCycler 480 system (Roche) in a 20 µL reaction containing 10 uL of SYBR Green I Master kit (Roche), 2 µl of cDNA (1/10 dilution of the first-strand reaction) and 200 nM of each primer. Each reaction was performed in triplicate. We quantified the glutamate decarboxylase 1 (*GAD1*); glutamate decarboxylase 2 (*GAD2*); sodium/potassium/chloride cotransporter 1 (*Nkcc1*), potassium/chloride cotransporter 2 (*Kcc2*); vesicular glutamate transporter 1 and 2 (*Vglut1/2*); Vesicular inhibitory amino acid transporter (*Viaat1, also known as Slc32a1 or Vgat*) expression using specific pairs of primers. Primer sequences are available upon request. The reference gene, Glyceraldehyde 3-phosphate dehydrogenase (Gapdh) was used to account for procedural loss. To normalize our results and quantify mRNA levels, we used the ^ΔΔ^C_t_ method [41]. After normalization statistical analysis was performed.

### Western Blotting

Caudate-putamen, hippocampal and ventral midbrain (SNpr+substantia nigra pars compacta) samples were micropunched as previously described. Samples were sonicated using a Bioruptor (Diagenode, Belgium) and proteins isolated in a lysis buffer containing 20 mM Tris-HCl pH = 7.5, 150 mM NaCl, 2 mM EGTA, 0.1% Triton X-100 and the complete protease inhibitor tablet (Roche). Protein concentration was determined using the BCA (Bicinchoninic acid) method. A denaturation step at 90°C for 5 min was applied only when testing GAD proteins. Proteins (20 µg) were separated on an 8% SDS-polyacrylamide gel and transferred onto a nitrocellulose membrane (Amersham Pharmacia Biotech) by liquid electroblotting (Mini Trans-Blot Cell, Bio-Rad) for 1 h at 200 mA. Non-specific binding was prevented by blocking the membrane with 5% nonfat dry milk in PBS 1× for 1 h at room temperature. Primary antibodies for GAD (GAD6, Developmental Studies Hybridoma Bank, 1∶500, mouse), NKCC1 (Developmental Studies Hybridoma Bank, 1∶1000, mouse), KCC2 (Millipore, 1∶1000, rabbit) and GAPDH (Santa Cruz, 1∶200, Goat) were diluted in 1% nonfat dry milk in PBS Tween 0.1% and incubated overnight at 4°C. After extensive washing of the membrane with PBS Tween 0.1%, membranes were incubated with the appropriate IRdye 800CW and 680RD secondary antibodies (LI-COR) for 2 h at room temperature. IRdye signals were visualised using the LICOR Odyssey Imager and images were exported as RGB tif files. Densitometric analysis was performed using the ImageJ software (NIH) and the values obtained for the gene of interest were normalized to GAPDH values used as a loading control. After normalization statistical analysis was performed.

### In vivo tiagabine treatment of *Mecp2*-deficient mice

From P30, animals received a tiagabine treatment through their drinking water (10 mg/kg/day) (Cephalon Inc., Pennsylvanie, USA). The dose was chosen in accordance with previous reports [42]–[44]. A total of twenty three *Mecp2*-deficient mice were studied (n = 10 tiagabine-treated and n = 13 untreated). Every other day, the volume of solution drunk by each mouse from calibrated pipettes was carefully measure. Adding tiagabine to the drinking water did not affect average water consumption that was 4.2±0.3 ml for untreated and 4.0±0.2 ml/day for the tiagabine-treated mice at P30, for instance. Throughout the postnatal development period, we modified the dose of tiagabine added to the drinking water accordingly to the body weight and to the liquid consumption. At the end of the study, we calculated that tiagabine consumption was between 8.78 mg/kg/day and 11.33 mg/kg/day. We have also assessed the effect of the tiagabine on the *Mecp2*-deficient mice treatment using behavioral tests (rotarod, open field, grip strength test), as previously described ([9]–[11], [45]). Briefly, the locomotor activity was measured in an open-field arena made of clear Perspex (ø100 cm). The test session lasted 20 min, and animal movements were recorded using the Viewpoint tracking system (Viewpoint SA Technology). Velocity (cm/s) and total distance moved (cm) were recorded. Velocity calculations were obtained using an input filter setting the minimal distance moved (0.5 cm) so that ambulations shorter than this value were never taken into account to calculate the velocity. A Bioseb grip strength meter (Panlab Technology) was used to measure the grip strength of mice. Two types of measurements were performed: forelimb measurement and forelimb and hind limb measurement. Five measures of each were taken and means were calculated from the three best trials.

### Statistical analysis

The data were analyzed using a one-way analysis of variance (ANOVA) followed by a Tukey's post hoc analysis using Prism 5.0 statistical software (GraphPad software, Inc.). Integrated ANOVA associate to a Bartlett's test, sensitive to departure from normality was used to verify the assumption of homoscedasticity. The results are expressed as mean ± standard error of the mean (S.E.M). The statistically significant *P*-values are shown as * *P*<0.05, ** *P*<0.01, and *** *P*<0.001.

## Results

### Postnatal changes in GABA and glutamate concentrations in specific brain areas of WT mice

**Figure 2:**
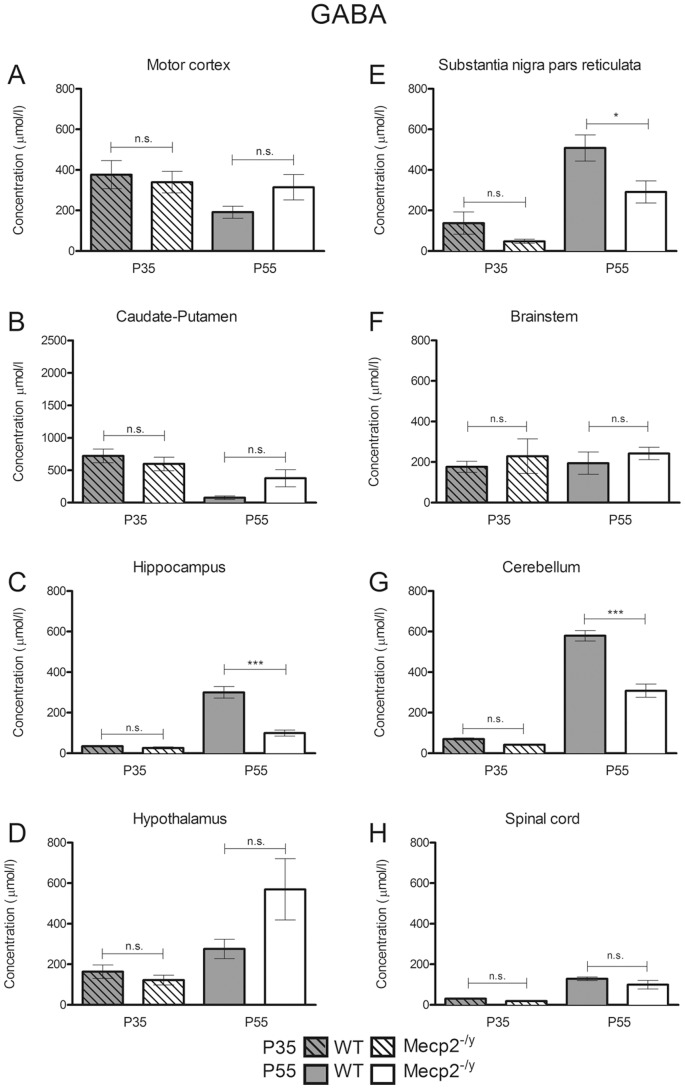
Neurochemical analysis of the GABA levels in A, the motor cortex; B, caudate-putamen; C, hippocampus; D, hypothalamus; E, substantia nigra pars reticulata; F, brainstem; G, cerebellum; H, spinal cord in P35 *Mecp2-*deficient (dashed/white bars) and WT (dashed/grey bars) (n = 6 *Mecp2^-/y^*, n = 9 WT for caudate-putamen, motor cortex, hypothalamus and brainstem/n = 6 *Mecp2^-/y^*, n = 6 WT for hippocampus, substantia nigra pars reticulata, cerebelum and spinal cord) and P55 *Mecp2^-/y^* (white bars) and WT (grey bars) mice dosage (n = 9 *Mecp2^-/y^*, n = 8 WT for motor cortex, hypothalamus and brainstem/n = 9 *Mecp2^-/y^*, n = 7 WT for caudate-putamen/n = 5 *Mecp2^-/y^*, n = 6 WT for hippocampus, substantia nigra pars reticulata, cerebellum, spinal cord). Results are expressed as mean ± S.E.M., (*P<0.05, **P<0.01, ***P<0.001).

**Figure 3:**
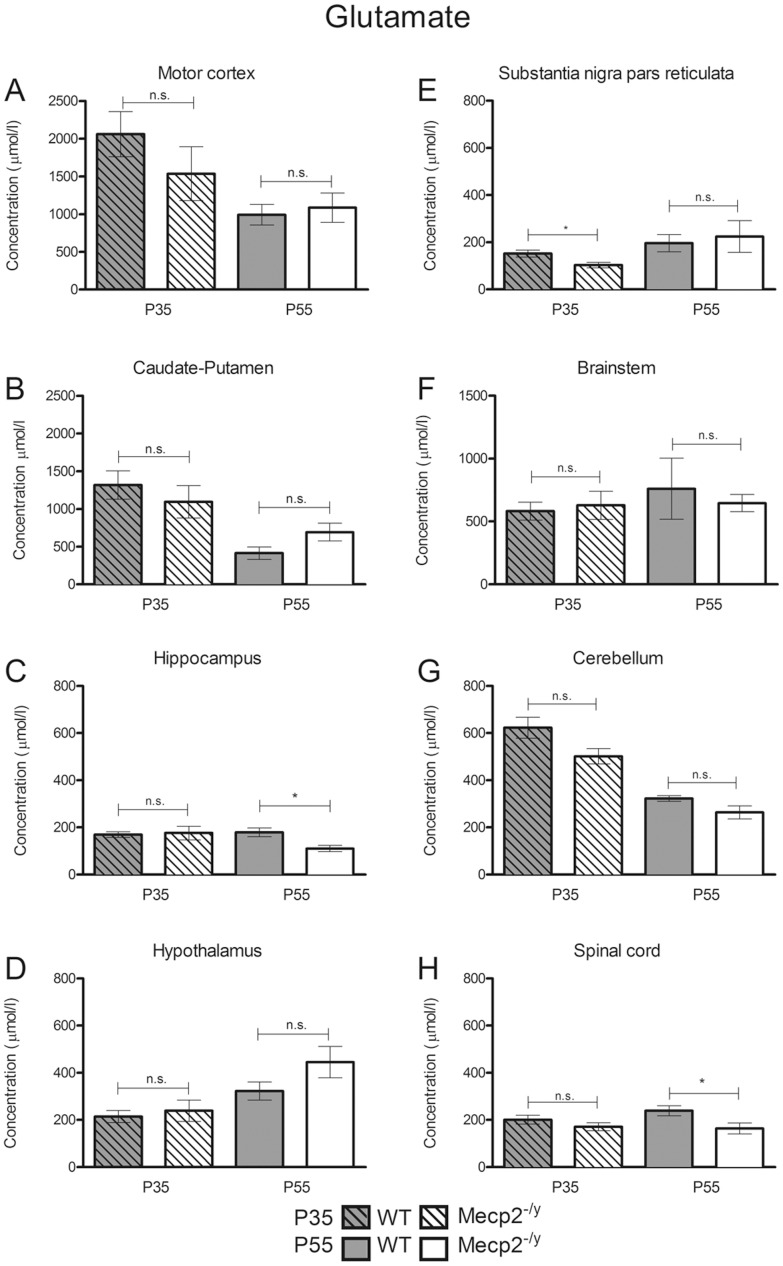
Neurochemical analysis of glutamate levels in A, the motor cortex; B, caudate-putamen; C, hippocampus; D, hypothalamus; E, substantia nigra pars reticulata; F, brainstem; G, cerebellum; H, spinal cord in P35 *Mecp2^-/y^* (dashed/white bars) and WT (dashed/grey bars) (n = 6 *Mecp2^-/y^*, n = 9 WT for caudate-putamen, motor cortex, hypothalamus and brainstem/n = 6 *Mecp2^-/y^*, n = 6 WT for hippocampus, substantia nigra pars reticulate, cerebellum and spinal cord) and P55 *Mecp2^-/y^* (white bars) and WT (grey bars) mice dosage (n = 9 *Mecp2^-/y^*, n = 8 WT for motor cortex, hypothalamus and brainstem/n = 9 *Mecp2^-/y^*, n = 7 WT for caudate-putamen/n = 5 *Mecp2^-/y^*, n = 6 WT for hippocampus, substantia nigra pars reticulata, cerebellum, spinal cord). Results are expressed as mean ± S.E.M., (*P<0.05, **P<0.01, ***P<0.001).

In the present study, the micropunch technique allowed us to restrict the neurochemical analysis to brain areas known to produce or integrate GABAergic and glutamatergic signals. We found that in WT mice, the GABA concentrations increased between P35 and P55 in the SNpr (+268.5%±51.4, *P*<0.001), hippocampus (+765%±91.3, *P*<0.001), cerebellum (+726%±40.2, *P*<0.001) and spinal cord (+313%±30, *P*<0.001) (Figure 2, Table 1) while GABA level progressively decreased in the caudate-putamen (−89.5%±4.1, *P*<0.01). In the other areas (motor cortex, hypothalamus, brainstem), GABA levels remained unchanged (*P*>0.05). The glutamate levels decreased between P35 and P55 in the motor cortex (−51%±7, *P*<0.01), caudate putamen (−68.6%±6.7, *P*<0.01) and the cerebellum (−48.2%±2.1, *P*<0.001) (Figure 3, Table 2) while they remained unchanged (P>0.05) during the same period in the other studied areas (hippocampus, hypothalamus, SNpr, brainstem, spinal cord).

**Table 1:** Developmental changes (P55 vs P35) in GABA concentrations in *Mecp2*-deficient or wild type mice.

	Genotype	
Structure	Wild-type	*Mecp2* ^-/y^
Motor cortex	n.s	n.s
Caudate-Putamen	**↓** (*P*<0.01)	n.s
Hippocampus	**↑** (*P*<0.001)	**↑** (*P*<0.05)
Hypothalamus	n.s	**↑** (*P*<0.05)
Substantia nigra pars reticulata	**↑** (*P*<0.001)	**↑** (*P*>0.05)
Brainstem	n.s	n.s
Cerebellum	**↑** (*P*<0.001)	**↑** (*P*<0.001)
Spinal cord	**↑** (*P*<0.001)	**↑** (*P*<0.001)

*Data for each group (WT or Mecp2^-/y^) was statistically analyzed by a one-way* ANOVA (P55 vs P35) and *P*-values are shown is parenthesis.

n.s, non-significant.

**Table 2:** Developmental changes (P55 vs P35) in glutamate concentrations in *Mecp2*-deficient or wild type mice.

	Genotype	
Structure	Wild-type	*Mecp2* ^-/y^
Motor cortex	**↓** (*P*<0.01)	n.s
Caudate-Putamen	**↓** (*P*<0.01)	n.s
Hippocampus	n.s	n.s
Hypothalamus	n.s	**↑** (*P*<0.05)
Substantia nigra pars reticulata	n.s	n.s
Brainstem	n.s	n.s
Cerebellum	**↓** (*P*<0.001)	**↓** (*P*<0.01)
Spinal cord	n.s	n.s

*Data for each group (WT or Mecp2^-/y^) was statistically analyzed by a one-way* ANOVA (P55 vs P35) and *P*- values are shown is parenthesis.

n.s, non-significant.

### Effect of *Mecp2* deletion on the GABA and glutamate concentrations in different mouse brain regions

We focused our study on several discrete brain regions known to contribute to the *Mecp2*-deficient phenotype in order to determine whether or not the GABA and glutamate levels were affected in the *Mecp2*-deficient mice brain. The results (Figure 2) showed that whatever the structure considered, the GABA levels were unaffected at pathology onset (P35) (*P*>0.05) when comparing *Mecp2*-deficient mice to their WT controls. Contrary to what was observed at P35, the GABA levels were significantly reduced in the hippocampus (−66.9%±5.5, *P*<0.001), the substantia nigra pars reticulata (−42.6%±11.9, *P*<0.05) and the cerebellum (−46.8%±6.3, *P*<0.001) at P55, when the mice are severely affected. Interestingly, despite a tendency toward an increase in the GABA concentrations at P55 in the motor cortex (+64.2%±34.5, *P*>0.05), the caudate-putamen (+398%±185, *P* = 0.069) and the hypothalamus (+6.8%±58, *P*>0.05), those changes remained statistically non-significant. Finally, GABA levels in caudal territories such as the brainstem and the spinal cord did not differ (*P*>0.05) in fully symptomatic (P55) *Mecp2*-deficient mice from their WT littermates.

Concerning the glutamate dosage (Figure 3), our results showed that this excitatory amino acid is found at similar concentrations in *Mecp2*-deficient and WT mice in most of the selected brain areas (motor cortex, caudate-putamen, hypothalamus, brainstem, cerebellum, *P*>0.05 in all structures) at both ages (P35, P55). The dosage revealed, however, a significant decrease in glutamate content in the *Mecp2*-deficient mice hippocampus (−38.3%±8.2, *P*<0.05) and spinal cord (−31.5%±11, *P*<0.05) at P55 only, following disease progression. In the SNpr the shift goes in the opposite direction with a significant reduction in glutamate concentration at P35 (−32%±8.4, *P*<0.05) while no significant difference was found at P55 (+14%±38.5, *P*>0.05).

### Abnormal expression of genes involved in the GABAergic and glutamatergic metabolism in the brain of *Mecp2*-deficient mice

**Figure 4:**
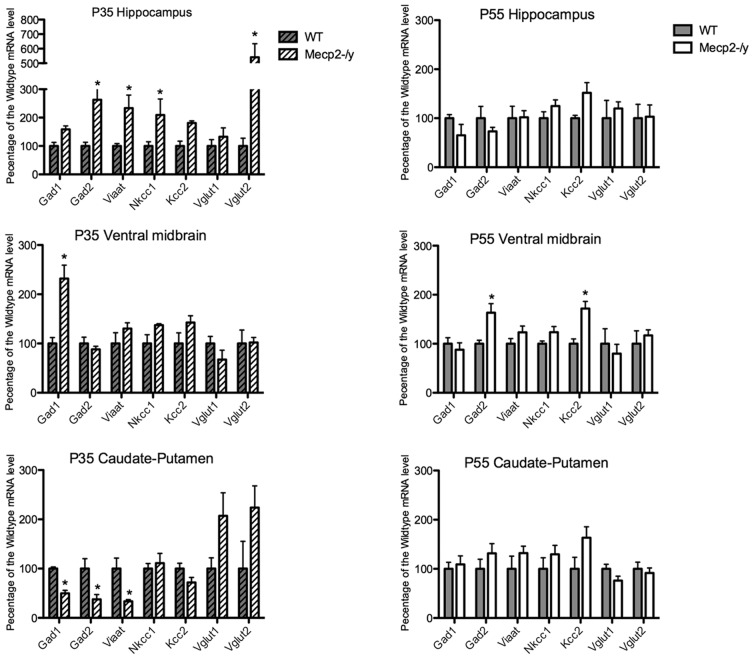
Relative expression level of genes involved in the GABA and glutamate metabolism in three brain areas, using quantitative RT-PCR. RNA samples were obtained from either WT or *Mecp2* ^-/y^ mice at two different ages (P35: *Mecp2^-/y^* t dashed/white bars and WT dashed/grey bars and P55: *Mecp2*-*^-/y^* t white bars and WT grey bars). The y-axis shows gene expression levels relative to wild type. Wild type level was arbitrarily set to 100%. Gene expression levels were normalized using GAPDH expression (*P<0.05, **P<0.01).

We next sought to determine whether the changes observed in GABA and Glutamate contents were mirrored by changes in their respective metabolic pathways We therefore quantified the mRNA brain expression of several key genes involved in the GABAergic and glutamatergic metabolism in non-symptomatic (P35) and symptomatic (P55) *Mecp2*-deficient mice and their wild-type littermates. We studied the expression of 7 genes : (I) the glutamic acid decarboxylase 1 (*GAD1* also called *GAD*67), (II) the glutamic acid decarboxylase 2 (*GAD2* also called *GAD65*), involved in the biosynthesis of the GABA, (III and IV) the vesicular glutamate transporter 1 and 2 (*Vglut1/2*) and (V) the vesicular inhibitory amino acid transporter (*Viaat1*, also known as Slc32a1 or *Vgat*), involved in the vesicular loading of glutamate and GABA respectively and finally, two membrane cotransporters, (VI) sodium potassium chloride cotransporter 1 (*Nkcc1*) and (VII) potassium chloride cotransporter 2 (*Kcc2*) that regulate the chloride driving force, thus altering the GABA polarity (excitation/inhibition) in response to GABA-A receptor activation (for review[46]). Among the different microdissected areas used for chromatographic analysis, we selected the caudate-putamen, the hippocampus and the ventral midbrain (substantia nigra pars reticulata+pars compacta). Our results (Figure 4, Table 3) show that in the caudate-putamen, changes in *Mecp2*-deficient mice were mainly marked by a downregulation of GABAergic markers such as *GAD1* (−50.1%, *P*<0.05), *GAD2* (−62.4%, *P*<0.05) and *Viaat1* (−66.3%, *P*<0.05) at the early stage (P35) and no difference later on. The other GABAergic (*Nkcc1*, *Kcc2*) and glutamatergic markers (*Vglut1*, *Vglut2*) did not significantly change at any stages. On the contrary, in the hippocampus, GABAergic markers including *GAD2* (+162.9%, *P*<0.05), *Viaat1* (+133.5%, *P*<0.05) and *Kcc2* (+80.9%, *P*<0.05) were mainly upregulated, whereas the glutamatergic marker *Vglut2* showed an increase only at P35 (+441.1%, *P*<0.05), with no changes later on. Our neurochemical dosages revealed that GABA levels were significantly reduced in the hippocampus of symptomatic *Mecp2*-deficient mice. Looking at a lower level of integration, we observed an opposite regulation of GABAergic marker transcripts with a consistent upregulation of *GAD2*, *Viaat1* and *Kcc2* at the early stage of the disease. If these results appear counterintuitive, it also suggests that hippocampal *Mecp2*-deficient neurons are able to maintain a normal level of GABA at P35 but fail to sustain this level of production at P55, when GABAergic metabolic transcripts expression is no longer increased. These results also raise the point that not only the key GABAergic synthetic enzyme GAD2 is affected in this homeostatic response, but also the machinery involved in the vesicular incorporation of GABA and the chloride channels responsible for the maintenance of a mature electrophysiological response to GABA. In the midbrain area, there was an increase in *GAD1* (+132%, *P*<0.05) at P35, whereas *GAD2* (+63%, *P*<0.05) and *Kcc2* (+71.9%, *P*<0.05) were increased at P55.

**Table 3:** Expression changes of the key genes involved in the GABAergic and glutamatergic metabolism in the brain of pre-symptomatic and symptomatic in *Mecp2*-deficient versus wild type mice.

	Caudate-putamen		Ventral midbrain		Hippocampus	
mRNA	P35	P55	P35	P55	P35	P55
*GAD1*	**↓** (*P*<0.05)	n.s	**↑** (*P*<0.05)	n.s	n.s (*P* = 0.055)	n.s
*GAD2*	**↓** (*P*<0.05)	n.s	n.s	**↑** (*P*<0.05)	**↑** (*P*<0.05)	n.s
*Nkcc1*	n.s	n.s	n.s	n.s	n.s	n.s
*Kcc2*	n.s	n.s	n.s	**↑** (*P*<0.05)	**↑** (*P*<0.05)	n.s
*Viaat1*	**↓** (*P*<0.05)	n.s	n.s	n.s	**↑** (*P*<0.05)	n.s
*Vglut1*	n.s	n.s	n.s	n.s	n.s	n.s
*Vglut2*	n.s	n.s	n.s	n.s	**↑** (*P*<0.05)	n.s

*Data for each group (P35 or P55) was statistically analyzed by a one-way* ANOVA *(Mecp2^-/y^ vs WT)* and *P*-values are shown in parenthesis.

n.s, non-significant.

### The protein levels of key genes involved in the regulation of the GABAergic metabolism are mainly downregulated in the brain of *Mecp2*-deficient mice

Based on the mRNA results, we focused our attention on the GABAergic pathway and selected GAD, Nkcc1 and Kcc2 to study their protein expression levels. Our results (Figure S1, S2, S3, 5, Table 4) showed that in caudate-putamen of *Mecp2*-deficient mice GAD decreased at P35 only (−51%, *P*<0.05) in accordance with the reduced mRNA levels of *GAD1* and *GAD2*. Strikingly, the reduction of the GAD levels at P35 did not result in a reduction of the GABA concentration at P35 when the symptoms are not too severe. The ventral midbrain showed a decrease in GAD at P55 (−38%, *P*<0.05) that could explain the reduction of GABA levels at this symptomatic age. Finally, the changes were more pronounced in the hippocampus where a decrease in GAD proteins was observed at P35 (−46%, *P*<0.05). Kcc2 protein levels were decreased at both P35 and P55 (−27%, *P*<0.05; −36%, *P*<0.05, respectively) mirroring the decreased GABA levels at P55. As for the mRNA expression study, we found evidence of deregulation in the expression of GABAergic-related proteins in *Mecp2*-deficient mice. Although these changes in mRNA levels are not closely correlated to the ones observed at the protein level, the picture that emerges clearly indicates that the *Mecp2* deficiency leads to functional deregulations in the two major brain neurotransmitter pathways in a temporally- and spatially-dependent manner.

**Figure 5:**
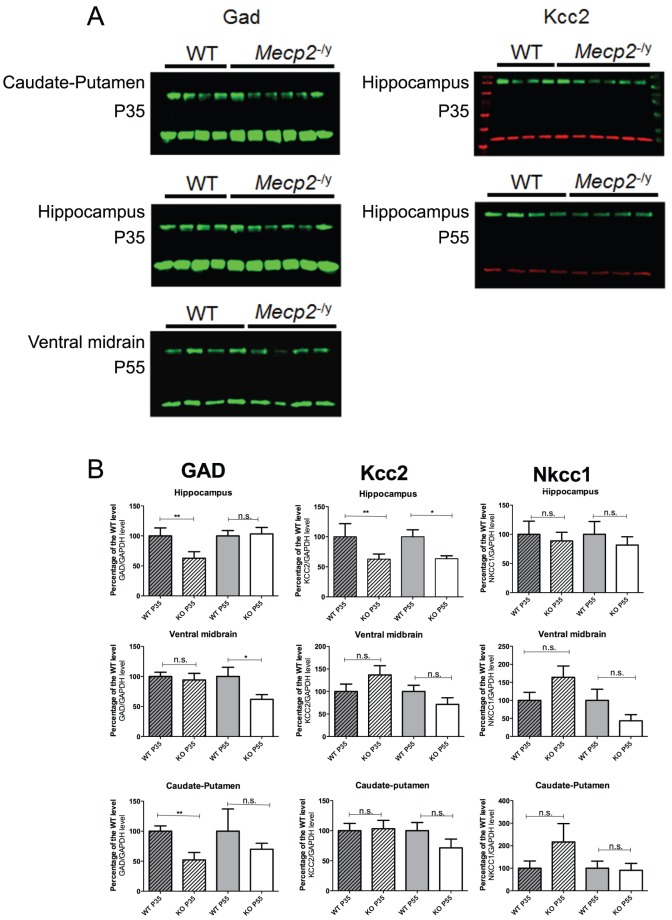
Alteration of the GABA/glutamate pathway in the *Mecp2* brain, at both late and early stages of the disease. A) Examples of western blots showing significant differences between *Mecp2^-/y^* and WT samples. On the left: Western blot analysis of GAD enzymes (GAD1, GAD2) in the caudate-putamen and hippocampus at an early stage (P35) and in the ventral midbrain at a late stage of the disease (P55). Each lane represents a sample from a different animal. On the right: Western blot analysis of the potassium-chloride transporter member 5 (Kcc2 or Slc12a5 in the hippocampus where a reduction of this channel is significant at an early (P35) and late stage. Each lane represents a different animal B) Quantification of all western blots carried out in the study, at both stages (P35: *Mecp2^-/y^* dashed/white bars and WT dashed/grey bars and P55: *Mecp2^-/y^* white bars and WT grey bars) in three structures of the brain (hippocampus, Caudate-putamen and Ventral Midbrain). Levels of the three proteins of interests (GAD2, Kcc2, Nkcc1) were normalized to GAPDH protein level. For all panels, n = 4–6 animals/group. Results are expressed in percentage relative to wild type animals arbitrarily set to 100%. (*P<0.05, **P<0.01).

**Table 4:** Protein changes during development in *Mecp2*-deficient and wild type mice.

	Caudate-putamen		Ventral midbrain		Hippocampus	
Protein	P35	P55	P35	P55	P35	P55
GAD	**↓** (*P*<0.01)	n.s	n.s	**↓** (*P*<0.05)	**↓** (*P*<0.05)	n.s
Nkcc1	n.s	n.s	n.s	n.s	n.s	n.s
Kcc2	n.s	n.s	n.s	n.s	**↓** (*P*<0.05)	**↓** (*P*<0.05)

*Data for each group (P35 or P55) was statistically analyzed by a one-way ANOVA (Mecp2^-/y^ vs WT) and P-values of the ANOVA during developmental changes are shown in parenthesis.*

n.s, non-significant.

### Stimulation of GABA signaling extends the lifespan of *Mecp2*-deficient mice

**Figure 6:**
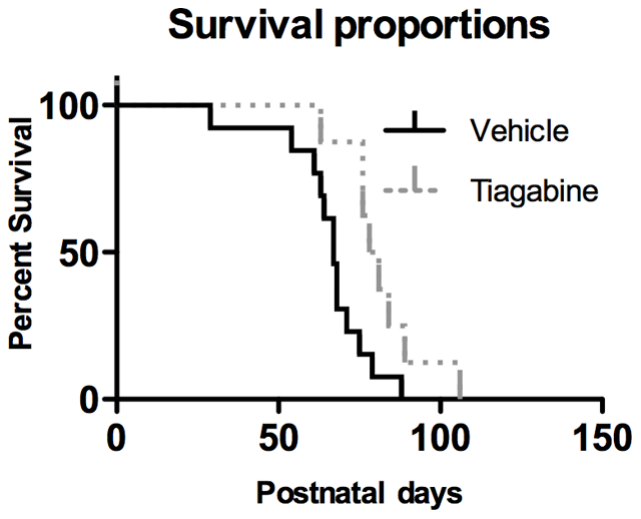
Chronic treatment with the GABA reuptake inhibitor tiagabine significantly extends the lifespan of *Mecp2*-deficient mice. The lifespan was measured in animals treated with the vehicle (drinking water) or tiagabine. The vehicle group lived 67.1±3.3 days (n = 13) while the tiagabine-treated group lived for 79.5±4.7 days (n = 10). The survival analysis was performed using a Kaplan-Meir log-rank test (*P*<0.05).

**Figure 7:**
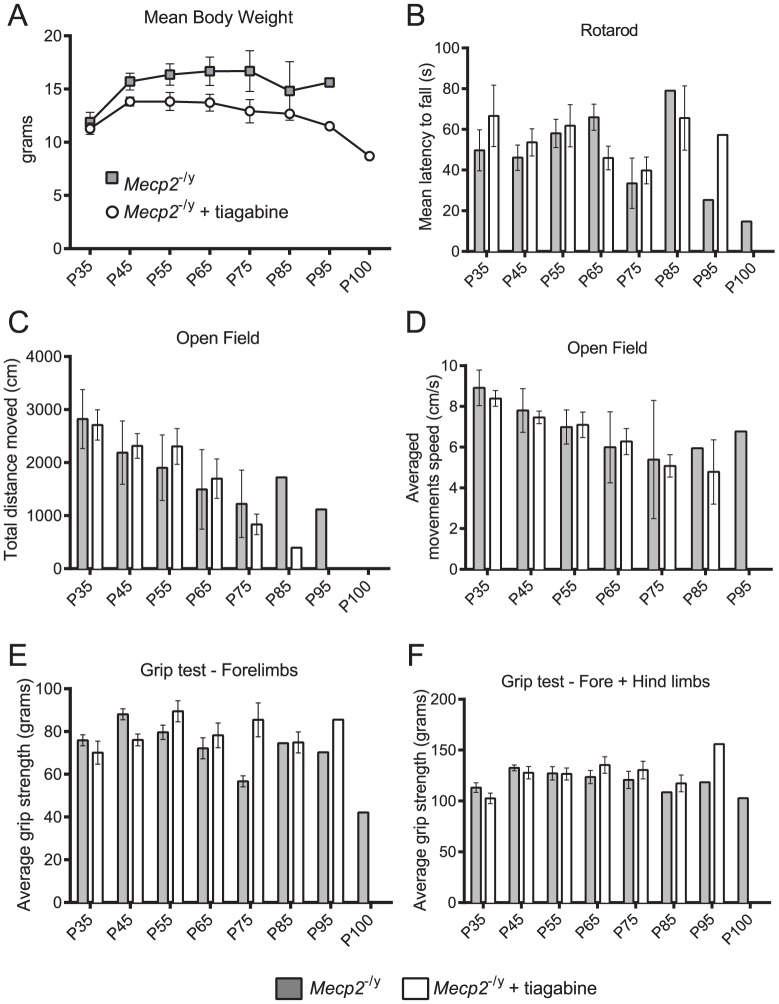
Impact of the oral tiagabine treatment on the motor performances of *Mecp2* ^–/y^ mice (Tigabine treated n = 10 and untreated n = 13 at P30). Histograms showing the body weight and the behavioral performances in the tiagabine-treated (*Mecp2* ^–/y^ Tiagabine, gray) and the vehicle group (*Mecp2* ^–/y^ vehicle, black) animals. The body weight curve starts from P30 to P100, and there were no significant differences between treated and untreated *Mecp2* ^-/y^. Rotarod: *Mecp2* ^–/y^ tiagabine (n = 10) and *Mecp2* ^–/y^ vehicle (n = 13) performed similarly at each developmental stages. Open field test: The total distance traveled by the *Mecp2* ^–/y^ mice and their average velocity were not affected by tiagabine treatment at any developmental stage. Grip strength test: The forelimb and forelimb+hind limb grip strength measurements were unaffected by the tiagabine treatment at all ages. Data are shown as mean±SEM.

Provided that the majority of the observed deregulations affect the GABAergic pathway, we evaluated the efficacy of a treatment stimulating the GABAergic metabolism in *Mecp2*-deficient mice. Tiagabine (Gabitril®), a new generation anti-epileptic and FDA-approved drug was selected because of its capacity to enhance the GABAergic activity through an inhibition of the GABA reuptake pathway [42]–[44]. Tiagabine binds to the GABA uptake carrier and potentially blocks the GABA uptake into presynaptic neurons, permitting more GABA to be available for binding to post-synaptic cell surface receptors. Our results (Figure 6) show that a chronic oral treatment with tiagabine hydrochloride given in drinking water (vehicle) at a concentration of approximately 10 mg/kg/day, extended the lifespan of *Mecp2*-deficient mice (79,5±4,7 days) compared to their *Mecp2*-deficient littermates treated with vehicle only (67,1±3,3 days) (*P*<0.05, Kaplan-Meir log-rank test). Using a set of behavioral tests, we evaluated the potential therapeutic effect of tiagabine treatment on motor function parameters in *Mecp2*-deficient mice (Figure 7). None of the selected tests allowed us to highlight any *in vivo* treatment improvement. The forelimb/hind limb grip strength, the motor coordination (rotarod), the locomotion and the exploratory behaviors were neither positively nor negatively affected by tiagabine treatment.

## Discussion

In less than 10 years, Rett syndrome has become a prototypical model for the research in neurological diseases [47]. In this study, we have used the *Mecp2*-deficient mouse model in order to decipher the causes of the multiple neurochemical and behavioral deficits reported in RTT patients. Among those, bioaminergic deregulations have consistently been found in several brain areas of various RTT mouse models [9]–[12], [19], [24], [34]. Deregulations of the GABAergic system leading to a depression of GABA transmission in the brainstem and a subsequent increase in excitation in caudal areas have also been reported [21]–[24]. Conversely, a decrease of the glutamatergic transmission was observed in the rostral territories [18], [19], [30], [31], a deficit that seems to originate in early development [17]. GABA and glutamate are the main neurotransmitters in the brain and are directly involved in a broad range of cognitive, autonomic and motor functions [48], [49]. *Mecp2* is highly expressed in GABAergic and glutamatergic neurons in the brain [50]. In line with the existence of an imbalance between excitation and inhibition in *Mecp2*-deficient mice and a role for GABA in RTT neuropathology, a recent work described how the selective removal of *Mecp2* in GABAergic neurons (*Viaat1*- and *Dlx5*-expressing cells) recapitulated key features of the *Mecp2*-deficient mouse pathology [51]. However, to the best of our knowledge, while several publications have documented a deregulation of theses two amino-acids in the whole brain or in large brain areas of *Mecp2*-deficient mice, no study has been reporting a detailed evaluation of the GABAergic and glutamatergic contents in specific parts of the brain. We therefore examined the role of Mecp2 in the regulation of the GABAergic and glutamatergic metabolic pathways in eight structures of the brain. The rationale that not all brain areas could equally be affected in the absence of *Mecp2* was motivated by the regional differences we found while investigating the bioaminergic system [9] and recently confirmed by Katz and colleagues [32]. Using multilevel approaches, we showed that *Mecp2* deficiency lead to a complex spatial and developmental deregulation of GABA and glutamate and some of their major metabolic synthetic enzymes and transporters.

### GABA and glutamate disturbances are not global

**Figure 8:**
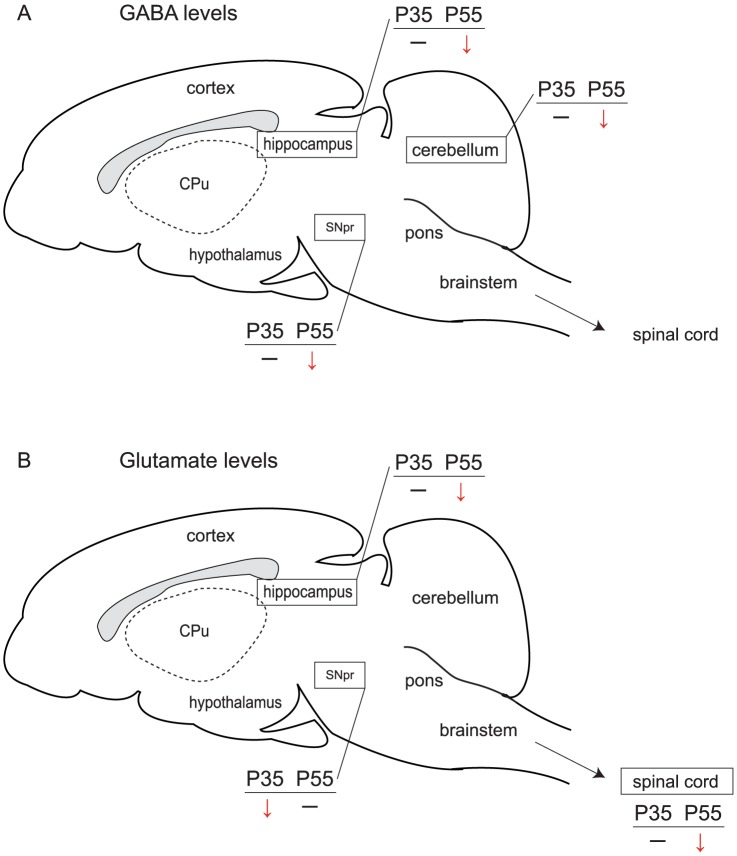
Summary of GABA (A) and glutamate (B) level measurements in the *Mecp2* ^-/y^ brain at P35 and P55. *Mecp2* deletion causes an age-dependent reduction of **A**) GABA levels in the hippocampus, the midbrain (SNpr, substantia nigra pars reticulata) and the cerebellum compared their WT littermates. The same trend was observed for **B**) the glutamate levels in the hippocampus and the spinal cord where the concentration was lower in *Mecp2^-/y^* compared to WT at P55. It is worth noting that glutamate is reduced in the SNpr at P35, a time corresponding to the onset of the mouse pathology. The sagittal mouse brain drawing is adapted from *The Mouse Brain in Stereotaxic Coordinates atlas* [38]. The gray area depicts the corpus callosum. CPu, Caudate-Putamen; SNpr, Substantia nigra pars reticulata.

One of the main results of our study was that, depending on the developmental stage, not all brain areas were similarly affected. This result was particularly interesting as it showed a differential effect of *Mecp2* deficiency on the main brain neurotransmitters. While the GABAergic and glutamatergic pathways were altered in a region- and developmental-specific manner, a global and progressive downregulation was seen in all key bioaminergic brain regions such as locus coeruleus or substantia nigra in the absence of *Mecp2* ([10], [11]). While not all brain regions were affected, all the changes seen in this study were a decrease in the GABA or glutamate content, with the hippocampus being the only structure showing a simultaneous decrease in both of them (Figure 8). It is noteworthy that in the present study we measured the global GABA and glutamate contents present in the different brain areas without any discrimination between intra- vs. extracellular levels or cell populations. In the light of our results it could be interesting now to use other methods such as microdialysis to evaluate the GABA or glutamate contents released at the synaptic level. For instance, the reductions of GABA levels were not correlated to glutamate compensatory increase. However, it was previously shown, using high-resolution magnetic resonance spectroscopy, that the glutamine/glutamate ratio is increased in *Mecp2-deficient* mice ([52]). In our study we did not follow glutamine synthetase, glutaminase and phosphodiesterase activities. These parameters should provide new insight to better understand the difference of amino-acid contents in the brain.

Glutamatergic and GABAergic neurons have been reported to directly affect the development of the disease [51], which could also involve deregulations in the GABA and glutamate contents, as seen in this study. However, most of the significant changes in GABA and glutamate contents in *Mecp2*-deficient mice observed here occurred at P55, when the phenotype is severe. One exception was the significant decrease of glutamate in the SNpr of P35 *Mecp2*-deficient mice. Therefore, we cannot rule out the possibility that the variations in GABA and glutamate seen here are a consequence rather than a cause of the disease.

### Protein and mRNA levels are differentially modulated but not tightly correlated – There is no GAD but GADs

Given the neurochemical changes seen in our study, we investigated the transcriptional and protein expression of genes involved in the GABA and glutamate pathways. Contrary to what we found in the neurochemical experiments, we only found transcriptional changes in the GABA-related genes, and mostly in early stage animals. It is important to note that our level of deregulation is moderated as seen in other studies. As reported by Chao et al [51], we found a significant decrease in the expression of the *GAD1* and *GAD2* mRNA in the striatum. When examined at the protein level, these same GABA-related genes showed expression changes that were not clearly correlated to the changes seen at the mRNA level, except for the caudate-putamen area.

This apparent discrepancy can result from a homeostatic response sustained by a functional redundancy between *GAD1* and *GAD2* and/or differential regulations masking changes occurring for only one isoform. The antibody used in the western blotting is expected to recognize GAD65 ([53], [54]) and does not probe possible GAD67 protein variations. The isoform-specificity represents a key factor since they preferentially account for different pool of GABA, *GAD2* (coding GAD65) being more representative of neurotransmission [55], [56].

Such uncoupling between mRNA and protein synthesis was also reported by Nguyen et al [57] and could be due to the defects in protein synthesis shown by Ricciardi et al [58] in another Rett mouse model.

Alternatively, we do the hypothesis that the hippocampus circuitry and its cellular diversity ([59], [60]) can also influence the level of expression of specific GABAergic genes. Depending on the cells considered (astrocytes, interneurons) and their correct integration in the hippocampus they could respond differently according to the level of neuronal activation or their position in an LTP/LTD-competent pathway. For instance GAD exists as two isoforms, GAD65 and GAD67, which are the products of two different genes (*Gad1* and *Gad2*) ([61]). Both *Gad* genes are coexpressed in the vast majority of GABA-positive neurons ([62]) but GAD65 seems to represent the most abundant GAD protein isoform in brain areas such as the dentate gyrus and the CA1 field in the rodent hippocampus ([63]). Immunohistochemichal studies with the same antibody (GAD6) we used for western blots experiments have shown that the GAD65 is localized in the nerve terminals, while GAD67, probed with the K2 antibody is expressed in mammalian neurons without specific subcellular enrichment ([54]). Indeed, data from the literature suggest that GAD65 is somatic and then transported along the axons toward the terminals to allow GABA synthesis and packaging into synaptic vesicles at the presynaptic level ([64]). Based on these observations and the limitations inherent to the methods we used (sampling and quantification), it is difficult to argue that any decrease/increase of GABA refers to any hippocampal field or is exclusively neuronal. As discussed previously we can also hardly relate those specific changes to intra- vs extracellular GABA contents.

### What about the glia?

Glutamate and GABA metabolic pathways are closely related and represent key steps in the regulation of synaptic transmission [65]. However, neurons are not the unique players in this phenomenon since glia, and in particular astrocytes, are part of the synaptic unit and modulate neurotransmitter availability through recycling and transport. For instance, astrocytes express excitatory amino acids transporters such as GLAST and GLT-1 and carry out the majority of the glutamate transport in the CNS [66]. In humans and rodents astrocytes GAD1, GABA transaminase and GABA receptors are expressed [67], [68]. These glial cells can also terminate the GABA neurotransmission by removing it from the extracellular milieu using the GAT1, GAT2 and BGT1 transporters [69].

Earlier studies suggested that RTT was due exclusively to the loss of Mecp2 function in neurons. Since then, it has been shown that the re-expression of *Mecp2* in astrocytes of *Mecp2*-deficient mice raised *in vitro* and *in vivo* parameters to normal level, and largely extended their lifespan compared to the *Mecp2*-deficient mice [70]. Therefore, from our data, we cannot exclude the influence of glia as all our samples contained both neuronal and glial cells. This information is of particular interest since Mecp2 has been found to affect astroglial genes expression in vitro and *Mecp2* deletion in astrocytes leads to an abnormal clearance of glutamate [71]. Moreover, it was reported that *Mecp2*-deficient microglia released a fivefold higher level of glutamate and that the blockade of microglial glutamate synthesis and release decrease the neurotoxicity in cell culture [72]. Further experiments will have to tease out the contribution of each cell compartment (neuronal/glial) in the deregulation of the amino acids metabolism in the context of *Mecp2*-deficiency.

### Tiagabine treatment improved lifespan in *Mecp2*-deficient mice

Compilation of our results and data published by other groups show that the fine-tuning of the amino acids metabolism by *Mecp2* is far from being fully explained. Nevertheless the GABA disturbances we identified through our multi-level approach appear complex and heterogeneous. A GABA deficit occurs at P55 in 3 brain areas of *Mecp2*-deficient mice, namely the hippocampus, the SNpr/ventral midbrain and the cerebellum, when these animals showed an overt phenotype. Since our neurochemical dosages mainly revealed a deficit in the *Mecp2*-deficient GABAergic system, we evaluated the potential therapeutic benefit of using a pro-GABAergic drug on these mice. In a previous study, Abdala et al. ([25]) have shown that combination of GABA (NO-711) and 5-HT1a reuptake blocker ameliorated both the breathing function and the lifespan of *Mecp2* heterozygous female mice. Moreover, L-838, a potent modulator of GABA-A receptors, also significantly reduced the number of apneas [25] in *Mecp2*-deficient mice. Several GABA enhancers have been used as antiepileptic drugs only, even though their benefit extend beyond epilepsy, ameliorating anxiety and various symptoms of neuropsychiatric disorders [73]. Their mechanisms of action range from GABA signal potentiation to modifications of GABA synthesis, metabolism or reuptake at the synaptic level [73]. Here, we showed that the GABA reuptake inhibitor tiagabine significantly extended the lifespan of the *Mecp2*-deficient mice. Despite to the lifespan improvement, we were not able to find any motor improvements under the tiagabine treatment. However, we cannot rule out that some other functions, not evaluated in this study, could be ameliorated. For instance, tiagabine is an antiepileptic drug, which could reduce this critical functional deficit often observed in RTT patient as well as in mouse model of RTT ([16]). Nevertheless, this result suggests that the modulation of the GABAergic pathway may be considered to alleviate some of the clinical signs. In addition, the availability of a wide range of GABAergic modulators already characterized and approved for clinical use by regulatory authorities (FDA, EMA) should allow for a faster translational usage.

## Supporting Information

Figure S1 **The GABA/Glutamate pathway and protein synthesis in the caudate-putamen in** ***Mecp2*** **^-/y^ and WT mice at an early and late stage of the disease.** Western blot analysis of caudate-putamen protein extracts from *Mecp2* ^-/y^ and WT mice at an early and late stage of the disease. Each lane represents a tissue sample from a different animal. Levels of proteins of interest were normalized to GAPDH protein level.
(PDF)

Figure S2 **The GABA/Glutamate pathway and protein synthesis in the hippocampus from** ***Mecp2*** **^-/y^ and WT mice at an early and late stage of the disease.** Western blot analysis of hippocampus protein extracts from *Mecp2* ^-/y^ and WT mice at an early and late stage of the disease. Each lane represents a tissue sample from a different animal. Levels of proteins of interest were normalized to GAPDH protein level.
(PDF)

Figure S3 **The GABA/Glutamate pathway and protein synthesis in the ventral midbrain from** ***Mecp2*** **^-/y^ and WT mice at an early and late stage of the disease.** Western blot analysis of ventral midbrain protein extracts from *Mecp2* ^-/y^ and WT mice at an early and late stage of the disease. Each lane represents a tissue sample from a different animal. Levels of proteins of interest were normalized to GAPDH protein level.
(PDF)
