# Supplementary figures and images for: GABA and Glutamate Pathways Are Spatially and Developmentally Affected in the Brain of Mecp2-Deficient Mice
Source: PLoS One. 2014 Mar 25;9(3):e92169. doi: 10.1371/journal.pone.0092169 (PMC3965407; doi:10.1371/journal.pone.0092169)

# Caudate -putamen

P35

P55

GAD

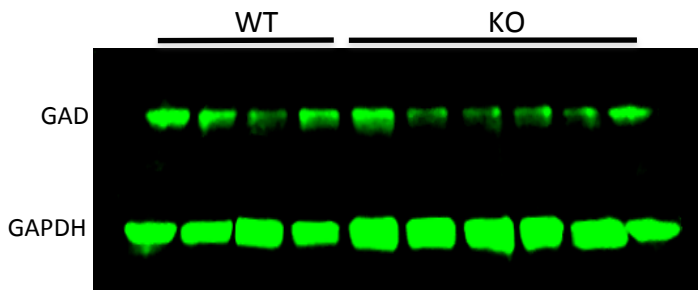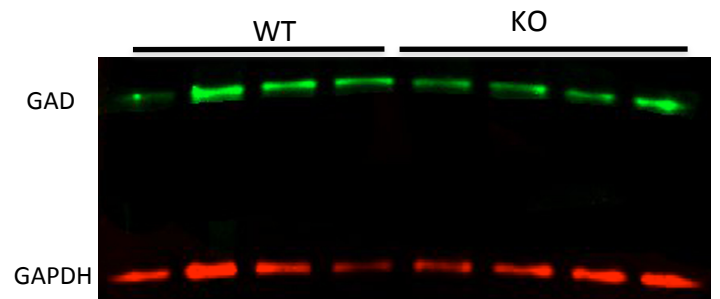

Kcc2

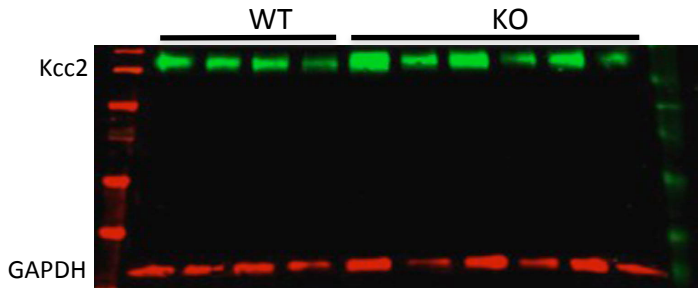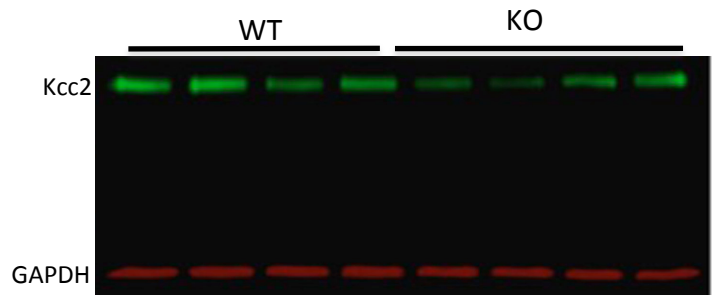

Nkcc1

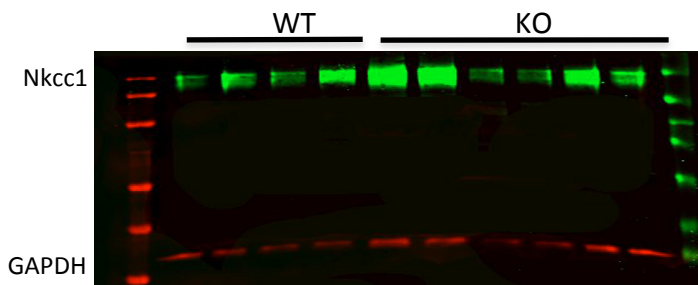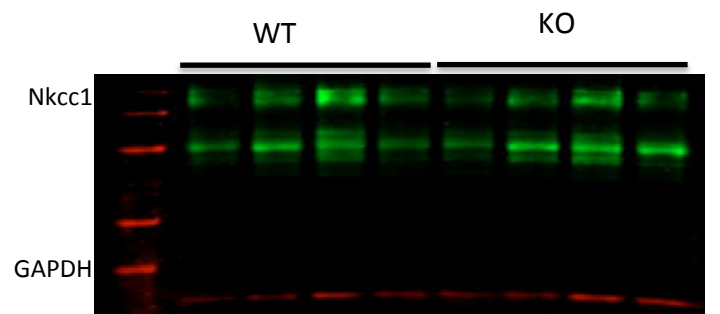

Supplement: Figure S1 — The GABA/Glutamate pathway and protein synthesis in the caudate-putamen in Mecp2 -/y and WT mice at an early and late stage of the disease. Western blot analysis of caudate-putamen protein extracts from Mecp2 -/y and WT mice at an early and late stage of the disease. Each lane represents a tissue sample from a different animal. Levels of proteins of interest were normalized to GAPDH protein level. (PDF) [file pone.0092169.s001.pdf]

Hippocampus

P35

P55

GAD

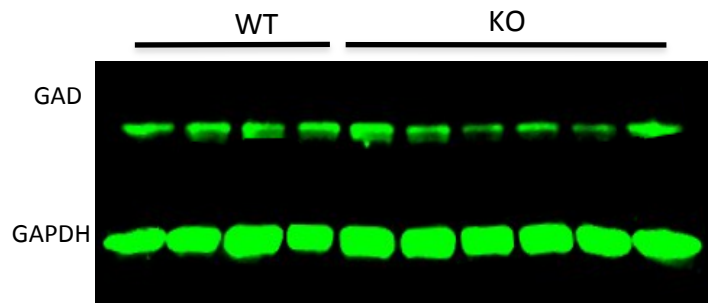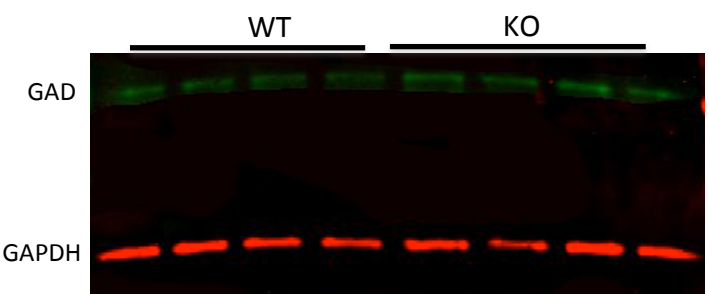

Kcc2

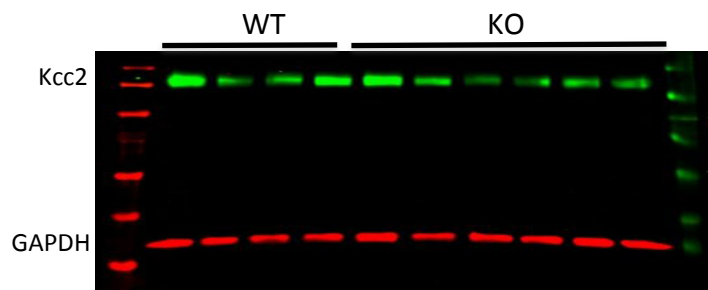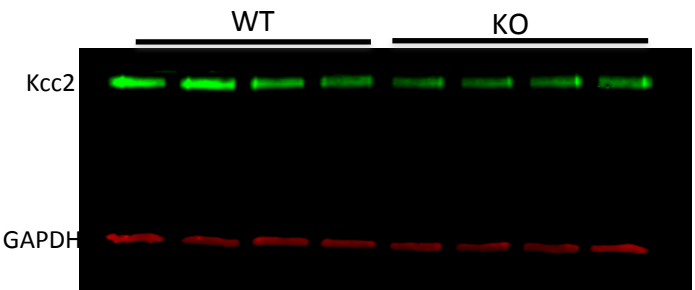

Nkcc1

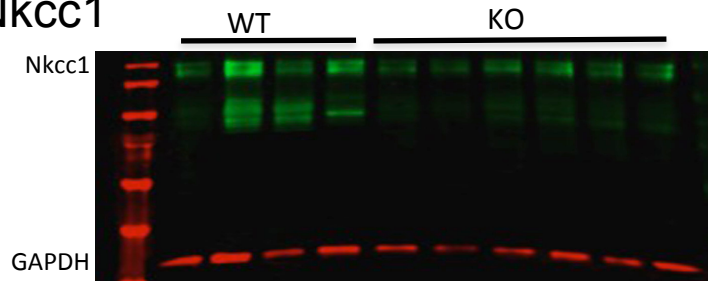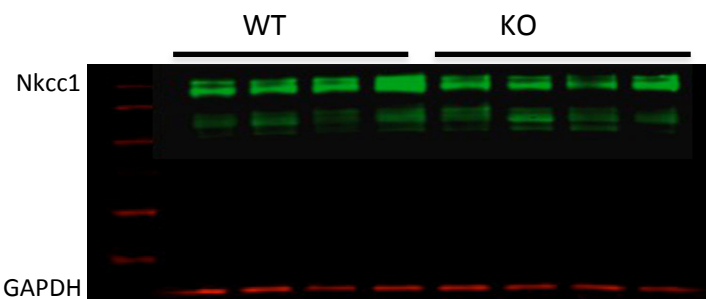

Supplement: Figure S2 — The GABA/Glutamate pathway and protein synthesis in the hippocampus from Mecp2 -/y and WT mice at an early and late stage of the disease. Western blot analysis of hippocampus protein extracts from Mecp2 -/y and WT mice at an early and late stage of the disease. Each lane represents a tissue sample from a different animal. Levels of proteins of interest were normalized to GAPDH protein level. (PDF) [file pone.0092169.s002.pdf]

# Ventral midbrain

## P35

### GAD

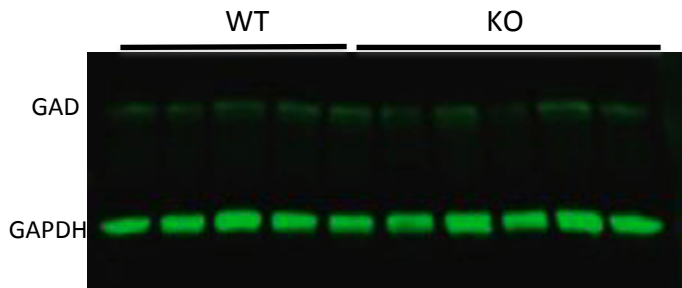

## P55

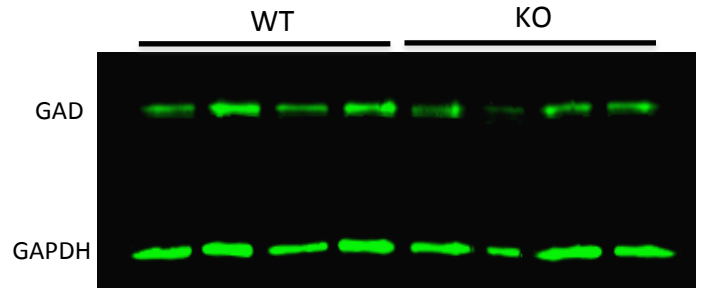

### Kcc2

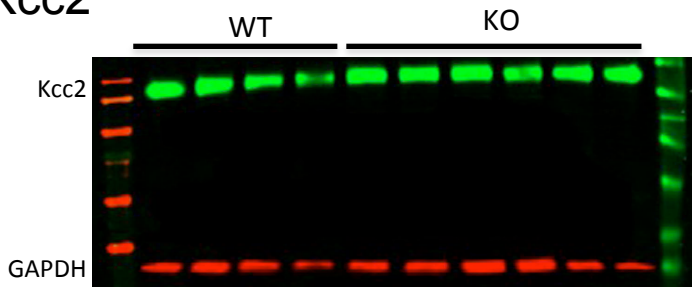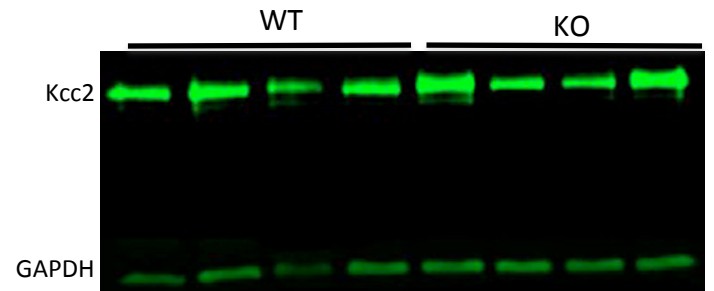

### Nkcc1

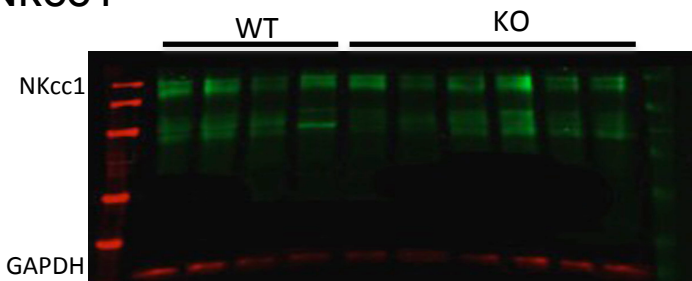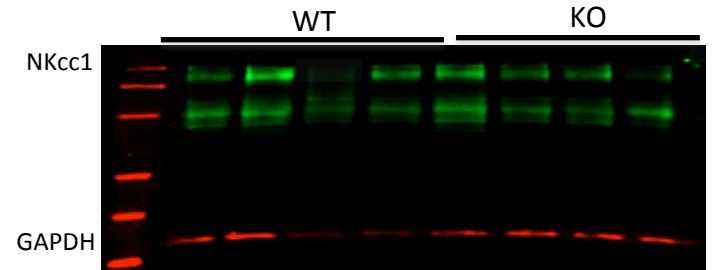

Supplement: Figure S3 — The GABA/Glutamate pathway and protein synthesis in the ventral midbrain from Mecp2 -/y and WT mice at an early and late stage of the disease. Western blot analysis of ventral midbrain protein extracts from Mecp2 -/y and WT mice at an early and late stage of the disease. Each lane represents a tissue sample from a different animal. Levels of proteins of interest were normalized to GAPDH protein level. (PDF) [file pone.0092169.s003.pdf]
